# Assessment of the Toxicity of Quantum Dots through Biliometric Analysis

**DOI:** 10.3390/ijerph18115768

**Published:** 2021-05-27

**Authors:** Lishi Zhong, Lili Zhang, Yimeng Li, Xue Liang, Lu Kong, Xiaobing Shen, Tianshu Wu

**Affiliations:** 1 Key Laboratory of Environmental Medicine and Engineering, Ministry of Education, School of Public Health, Southeast University, Nanjing 210009, China; liszhong@163.com (L.Z.); lilizhang2002@163.com (L.Z.); yimmmeng718@gmail.com (Y.L.); konglu_yaoyu@126.com (L.K.); 101006194@seu.edu.cn (X.S.); 2 School of Public Health, Shandong First Medical University (Shandong Academy of Medical Sciences), Jinan 250117, China; 230189319@seu.edu.cn

**Keywords:** nanotoxicology, bibliometrics, quantum dot, toxicity

## Abstract

Along with the rapid development of nanotechnology, the biosafety of quantum dots (QDs), a widely used kind of nanoparticles, has grabbed the attentions of researchers, because QDs have excellent and unique optical properties that other commonly used nanoparticles, like walled carbon nanotubes, do not have. The understanding of the toxicity of QDs is an important premise for their application in wider fields, including biology and medicine. This study sought to analyze scientific publications on the toxicity of QDs and to construct a bibliometric model for qualitative and quantitative evaluation of these publications over the past decade, which visually presented the status quo and future development trend on the toxicological study of QDs. A search for data using the triple blind method revealed that, as of 31 December 2018, there were 5269 papers published on the toxicity of QDs. RSC ADVANCES (5-year IF, 3.096) ranked first in the number of publications. China had the largest number of publications (2233) and the highest H-index (119), but the United States was still the leading country with regards to the quality of the research. LIU Y (106 publications) published the most papers, while Hardman R (304 co-citations) had the most co-citations. The keyword “walled carbon nanotube” ranked first in the research frontier. The findings not only determine a development trend of the toxicological study of QDs, but also identify further research directions in this field.

## 1. Introduction

Emerging nanotechnology can greatly improve the current medical status and provide targeted medical diagnosis and treatment, which could help solve many medical problems [1]. As a new concept proposed in the 1990s, Quantum dots (QDs) are a kind of commonly used nanomaterials, also called nanocrystals. A QD is a quasi-zero-dimensional nanomaterial with three dimensions less than 10 nm [2]. Generally, it is a nanocrystal composed of III-V or II-VI elements and comprises a semiconductor material core, along with an outer shell that increases the stability and/or biocompatibility of the core [3]. In recent years, many types of QDs have been synthesized. Various QDs exhibit different optical characteristics due to disparities in geometric shapes, quantum sealing effects of electrons and holes and materials, etc. [4].

As a new type of fluorescent nanomaterial, QDs have a wide range of applications in biomedicine and other fields owing to their excellent optical properties [5,6,7]. For example, QDs can participate in tumor tracking, microbial detection, and drug targeted therapy [8,9]. It is likely to become a core nanomaterial in the research of biomedical materials and has attracted wide attention from researchers around the world [10]. In recent years, more and more researchers have begun to explore QDs toxicity, toxic mechanism, and biosafety evaluation to assure their biosafety in clinical applications. Many researchers have found that QDs result in DNA damage, elevated levels of ROS (reactive oxygen species), and decreased cell viability [11,12]. At present, the well-known toxic mechanisms of QDs mainly include oxidative stress damage, inflammatory reaction theory, ion channel gating change damage, etc. [13,14]. A large number of research articles on the toxicity of QDs have been published in different academic journals. However, to the best of our knowledge, few researchers have tried to systematically analyze the evolution of the scientific output in this domain.

Recently, bibliometric analysis has been widely used to analyze published literature in one specific field, which helps assess the trend of research activities as time goes on. Research in the field of nanotechnology, such as on the risk or safety issues associated with nanomaterials and nanomedicine, have been reported to be analyzed through bibliometric analysis and have contributed to the development of this field [15,16,17]. The purpose of this study is to systematically evaluate the research on toxicity of QDs over the last ten years—from 2009 to 2018—using methods of statistics and mathematics in bibliometric analysis with the help of the CiteSpace V visual literature analysis software. The objectives of this study include ensuring the publication model of literature related to QDs’ toxicity, capturing the cooperation model between countries/institutions/authors, and confirming research trends and frontiers in this field. These findings provide valuable information on this topic in order to pioneer the field of toxicological study of QDs over the next few years.

## 2. Materials and Methods

### 2.1. Source of the Data and Search Strategy

**Figure 1:**
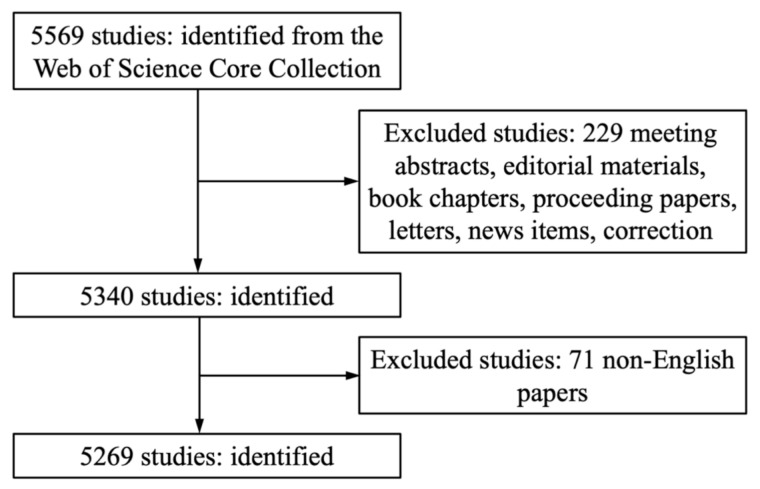
The process of retrieval and exclusion.

The Web of Science Core Collection (WoSCC), Thomson Reuters, was accessed on December 31, 2018, to search for literature on the toxicity of QDs. The WoSCC, including the Social Sciences Citation Index (SSCI) and the Science Citation Index-Expanded (SCI-E), provided detailed information on bibliometric analysis [18]. The terms used for data retrieval are listed in Supplementary Materials. The literature screening flowchart is shown in Figure 1.

### 2.2. Data Collection

All data were collected independently by the three first authors (Lishi Zhong, Lili Zhang, Yimeng Li) in order to avoid discrepancy of data as far as possible; the data were downloaded in TXT format. Data from the 5269 identified studies were imported into CiteSpace V (Drexel University, Philadelphia, PA, USA) and Microsoft Excel 2016 (Redmond, WA, USA) for qualitative and quantitative analysis and to export or draw figures and tables.

### 2.3. Statistical Methods

We used WoSCC to analyze the characteristics of all eligible publications, including journal sources, annual publications, contributing countries or territories, contributing authors, citation counts, H-indexes; the top 10 journals and top 10 countries/territories and institutions were chosen for summary in tables.

A model f(x) = ax + b was used to predict the temporal trend of publications with Microsoft Excel 2016 software (Redmond, WA, USA). The symbol x represented the year of publication and f(x) the cumulative number of publications by year.

All eligible documents were displayed through CiteSpace V (Drexel University, Philadelphia, PA, USA), which is a visualization software for analyzing data through network modeling [19]. CiteSpace V was used to: (i) identify cooperation between countries/territories/authors; (ii) capture relationships between different published journals; (iii) analyze the co-citation relationships between author/references; (iv) perform burst analysis of keywords.

## 3. Results

### 3.1. Publication Outputs and Growth Prediction

**Figure 2:**
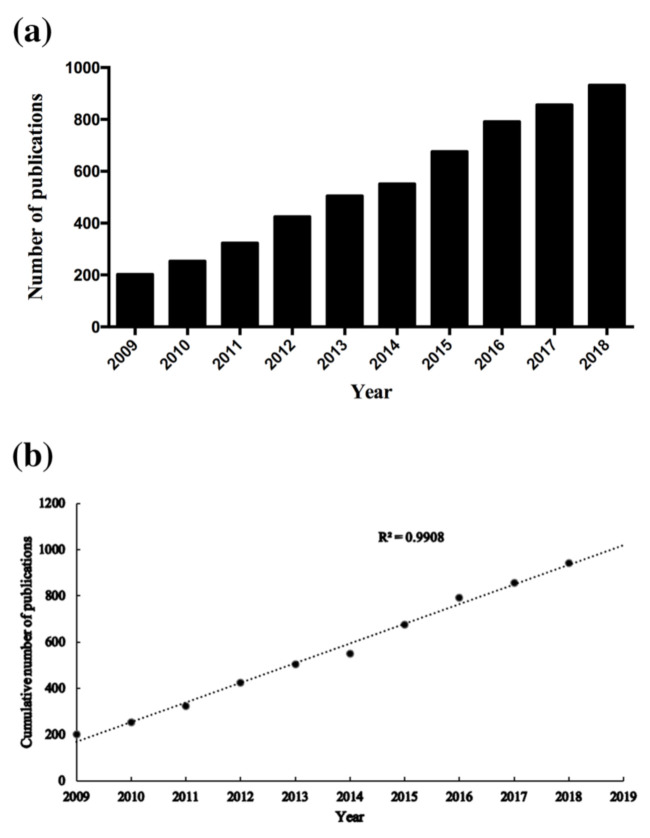
Publication outputs and growth prediction. (**a**) The number of annual publications on QDs toxicity research from 2009 to 2018; (**b**) The model-fitting curve of the growth trend of publications on QDs toxicity.

The distribution of annual publications in this domain for the period of 2009 to 2018 is summarized in Figure 2a. The number of publications kept persistently increasing with time—from 202 publications in 2009 to 932 publications in 2018.

The model-fitting curve for the number of publications on QDs toxicity research is presented in Figure 2b. It suggests that there exists a significant correlation between the cumulative number of publications and the publication year (R^2^ = 0.9908). By using the prediction model, the number of publications on the toxicity of QDs was estimated to reach 1016 in 2019. By the completion of our article, the total number of publications on QDs toxicity research in 2019 was 1072, which is basically consistent with our predictions using the prediction model curve.

### 3.2. Distribution by Journals

The 5269 publications on QDs toxicity research were published in 339 academic journals. Table 1 summarizes the top 10 journals that published articles in this domain. Among the top 10 journals, RSC Advances, the 5-year impact factor (IF) of which is 3.096, contributed most to publications on QDs toxicity research (237 publications, 4.37%). This was followed by Nanoscale (5-year IF, 7.713; 178 publications; 3.29%), ACS Applied Materials Interfaces (5-year IF, 8.284; 129 publications; 2.38%), and Biomaterials (5-year IF, 9.315; 126 publications; 2.33%).

When analyzing the IFs of the top 10 journals, only ACS NANO (5-year IF, 14.820) was found to have a 5-year IF higher than 10.000. Four of the journals had a 5-year IF between 5.000 and 10.000, including Biomaterials (5-year IF, 9.315), ACS Applied Materials Interfaces (5-year IF, 8.284), Nanoscale (5-year IF, 7.713), and Sensors and Actuators B-Chemical (5-year IF, 5.118). In addition, four of the journals had a 5-year IF between 3.000 and 5.000, including Journal of Materials Chemistry B (5-year IF, 4.959), Scientific Reports (5-year IF, 4.609), Nanotechnology (5-year IF, 3.467), and RSC Advances (5-year IF, 3.096). Moreover, journals with 5-year IFs higher than 3.000 contributed to 23.31% of the total publications. The contributions of journals with different 5-year IFs were as follows: 5-year IF > 10.000, 1.77%; 10.000 > 5-year IF > 5.000, 12.70%; 5.000 > 5-year IF > 3.000, 8.84%. In summary, it was extremely challenging to publish articles on QDs toxicity research in high-impact journals.

### 3.3. Distribution by Country and Institution

**Figure 3:**
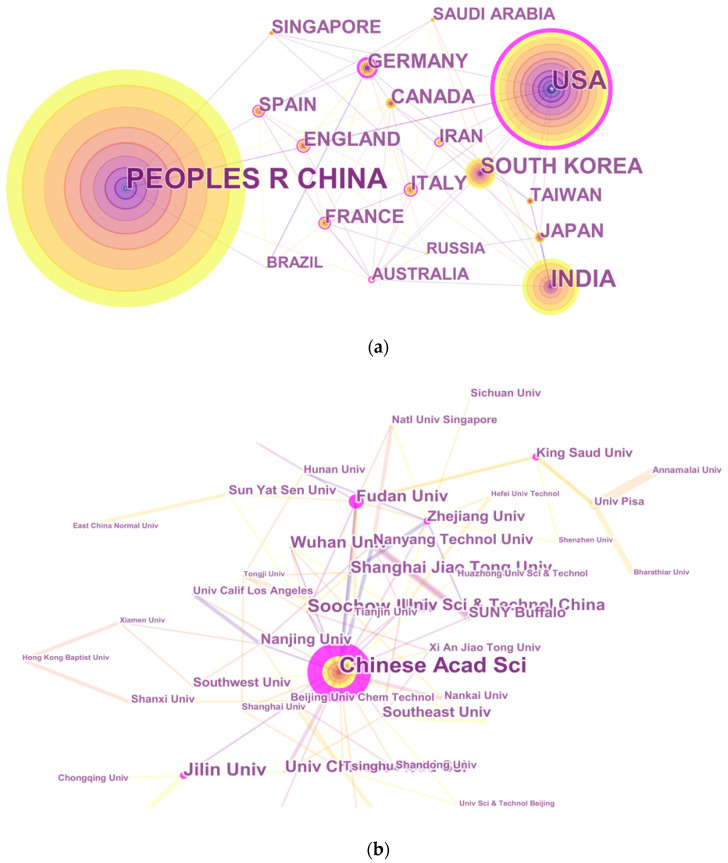
The analysis of countries/territories and institutions. (**a**) Network map of countries/territories engaged in QDs toxicity research; (**b**) Network map of institutions engaged in QDs toxicity research.

The 5269 articles on QDs toxicity research were provided by 88 countries. The network map in Figure 3a of countries/territories engaged in QDs toxicity research showed the existence of broad cooperation among these countries/regions. The top 10 countries/territories that contributed to QDs toxicity research (Table 2) included four from East Asia (China, India, South Korea, Japan), four from Europe (Germany, Italy, England, France), and two from North America (United States, Canada). China had the largest number of publications (2233), followed by the United States (1067), India (603), South Korea (349), and Germany (188).

Figure 3b presented the network map of institutions engaged in QDs toxicity research. More than 3800 institutions participated in publications on QDs toxicity research. The top 10 institutions accounting for 21.17% of the total number of publications were listed in Table 2; nine institutions (expect for Nanyang Technological University) were from China. Among these institutions, the Chinese Academy of Sciences led the first research echelon (368), followed by Jilin University (125) and Soochow University (103).

### 3.4. Analysis of Publications, Total Citations, and H-Index

**Figure 4:**
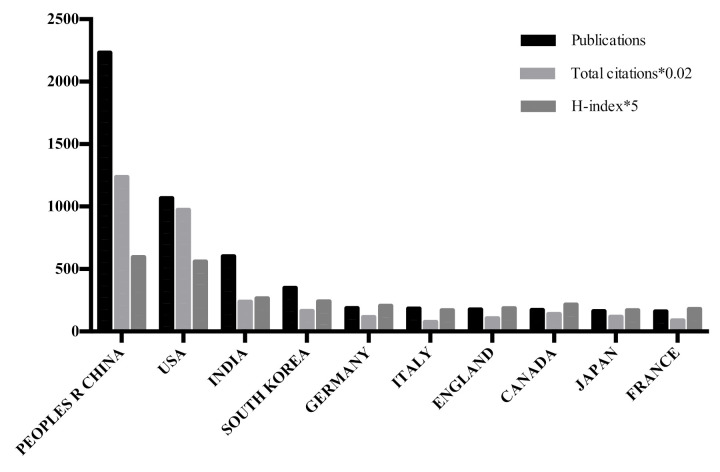
The number of publications, citations, and H-indexes on QDs toxicity research of the top 10 countries/territories.

The number of publications, total citations, and H-indexes from the top 10 countries/territories engaged in QDs toxicity research are shown in Table 1 and Figure 4. It is observed that China had both most citations (61,790) and the highest H-index (119). The United States ranked second both in citation frequency (48,664) and the H-index (112). It is worth noting that there are few differences in the ranking of these two items between other countries/territories, owing to the gap in the number of total publications.

### 3.5. Contribution by Authors

**Figure 5:**
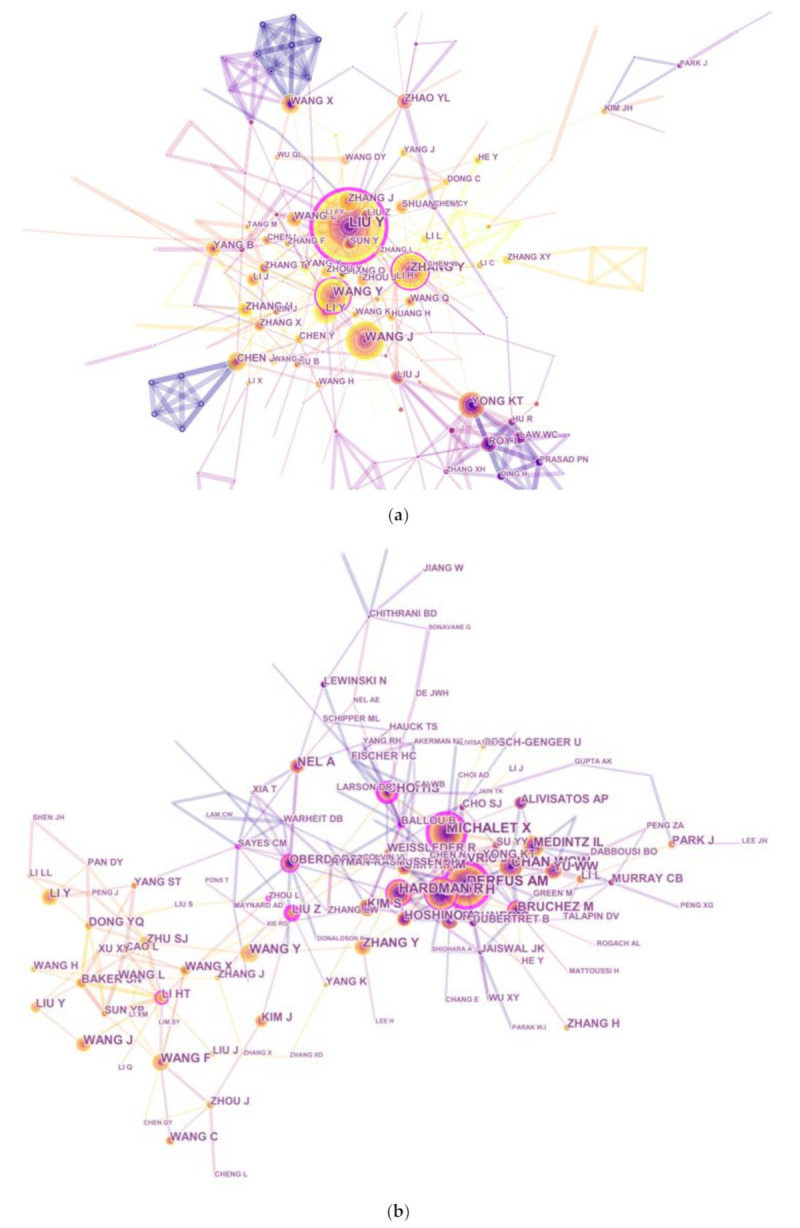
Analysis of the authors. (**a**) Network map of active authors who contributed to QDs toxicity research; (**b**) Network map of co-cited authors that contributed to QDs toxicity research.

Nearly 20,000 authors were engaged in QDs toxicity research for the studied period. The network map illustrates active authors who contributed to the publications in this domain and summarized the collaboration among authors (Figure 5a). The top 10 authors are listed in Table 3. As for the number of authors’ publications, LIU Y was first (106 publications), followed by WANG J (66 publications), ZHANG Y (61 publications), and WANG Y (56 publications). However, in terms of common citation counts, none of these prolific authors were included in the top 10 co-cited authors, suggesting that they should pay more attention to the quality rather than quantity of their publications. The top three co-cited authors had at least 430 co-citation counts, including Derfus AM (593 publications), Michalet X (492 publications), and Gao XH (432 publications).

Author citations were analyzed with the CiteSpace V software in order to estimate the relevance of scientific publications; the results are presented as a citation network in Figure 5b. Regarding the top 10 co-cited authors (Table 3), Derfus AM (593 citations) ranked first, followed by Michalet X (492 citations), Gao XH (432 citations), and Chan WCW (410 citations). Meanwhile, the top 10 co-cited references were considered to be the knowledge base in the field of QDs toxicity research. An article that Hardman R published in Environmental Health Perspectives (5-year IF, 9.837) had the highest co-citations (2006, 304 co-citations), followed by articles published by Michalet X (2005, 292 co-citations) in Science (5-year IF, 40.627), Derfus AM (2004, 266 co-citations) in Journal of Nano Letters (5-year IF, 14.201), and Baker SN (2010, 222 co-citations) in Angewandte Chemie-International Edition (5-year IF, 40.627).

### 3.6. Analysis of References

**Figure 6:**
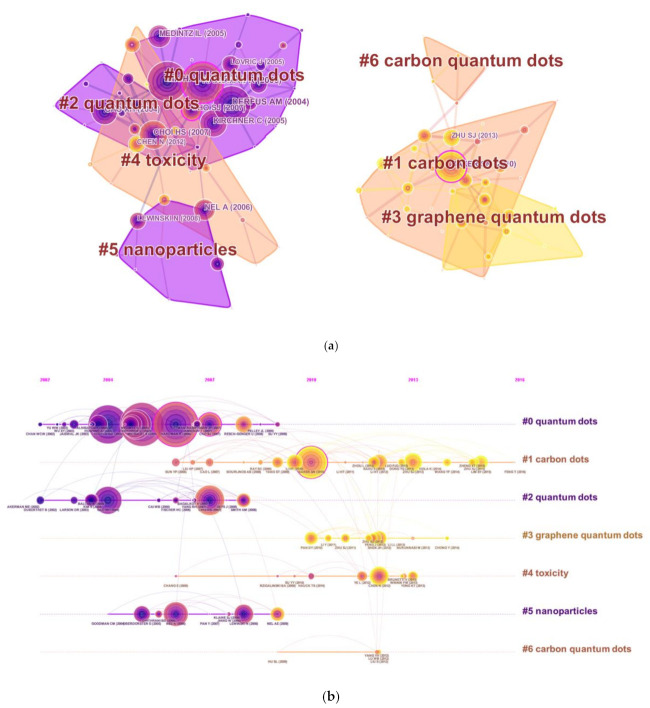
The analysis of references. (**a**) Co-citation map of references from publications on QDs toxicity research; (**b**) Co-citation map (timeline view) of references from publications on QDs toxicity research.

Reference analysis is regarded an important index in bibliometrics. Figure 6a presents the co-citation map of references from publications on QDs toxicity research, which manifested the scientific relevance of these publications in the field. All clusters were labeled with index terms extracted from the references. The largest cluster #0 was labeled as “quantum dots”, followed by the second largest cluster #1 labeled as “carbon dots” and the third largest cluster #2 labeled as “quantum dots”. These clusters are also illustrated in a timeline view (Figure 6b), which suggested the development tendency of QDs toxicity research.

### 3.7. Analysis of Keywords

**Figure 7:**
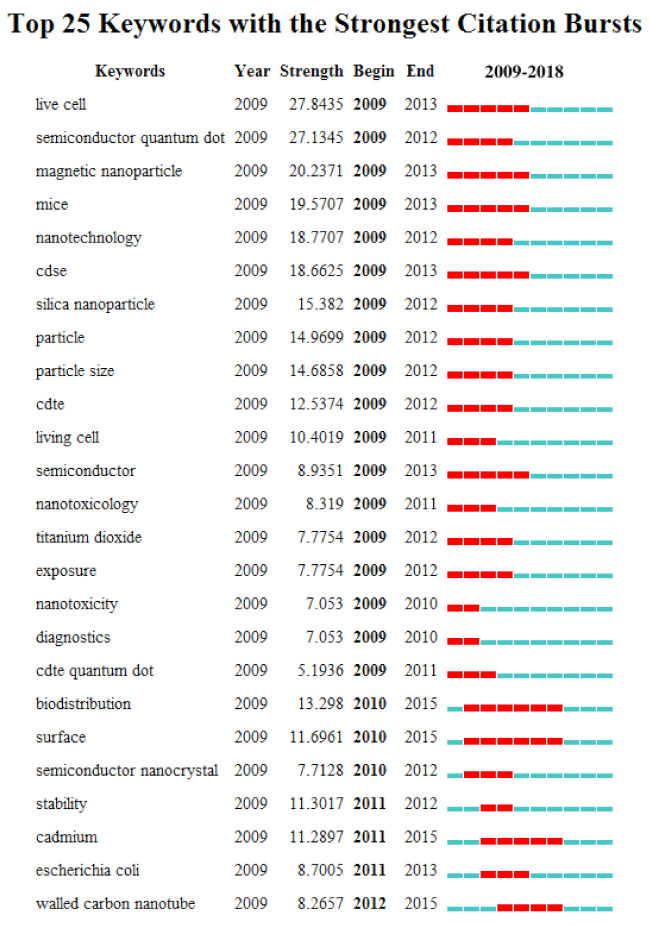
The keywords with the strongest citation bursts of publications on QDs toxicity research. The time intervals are plotted on the blue line, while the periods of burst keywords are plotted on the red line, indicating the beginning and end of the time interval of each burst.

CiteSpace V software was used to extract keywords from 5269 publications on QDs toxicity research. Furthermore, we detected and analyzed the strongest reference bursts using CiteSpace V software and the top 25 keywords with the strongest citation bursts; they are listed in Figure 7. The keywords most frequently cited since 2010 are: “surface” (2010–2015), “semiconductor nanocrystal” (2010–2012), “stability” (2011–2012), “cadmium” (2011–2015), “*escherichia coli*” (2011–2013), and “walled carbon nanotube” (2012–2015).

## 4. Discussion

According to the analyzed results, it is obvious that the number of publications on QDs toxicity research has continually increased over the last ten years; however, the growth rate of publications fluctuated over time. Based on the increased publications from 2009 to 2018, a predicted model curve was established and verified by the publication data in 2019. Based on this trend, we can infer that more research output will emerge. Therefore, we can expect developments in QDs toxicity research. In regards to countries/territories with extensive cooperation in the field of QDs toxicity research, China ranked first, followed by the United States and India, which indicates that China had made considerable progress in this domain. In addition, Chinese institutions accounted for the largest share of collaborative networks. This is why China contributed the most to publications related to QDs toxicity research. It is noted that the number of publications from the United States was less than half of that from China, while there is only a small gap regarding the total citations and H-index between the two countries, which suggests that the United States had shown its dominant position in those two aspects. Therefore, the United States is still the leading country ahead of China for quality of QDs toxicity research.

On deep investigation of the top 10 “prolific authors” listed in this study, we found that Liu et al. [20,21,22] mainly focused on the toxicity mechanisms of different functional cadmium-containing QDs as well as the functional research of novel carbon dots. Wang et al. [23,24] engaged in the preparation of new carbon dots and evaluated the toxicity of the prepared carbon dots. Publications by Zhang et al. [25,26] emphasized on the development of new QDs materials and the biological tracing of these new materials. Among these 10 authors, two of them were included in the top 10 co-cited authors, which indicated that they played an important role in QDs toxicity research. Among the top 3 co-cited authors listed here, i.e., Derfus AM, Michalet X, and Gao XH, studies by Derfus AM showed that the cytotoxicity of QDs was associated with the release of free Cd^2+^ ions, and the cytotoxicity of QDs was modulated by processing parameters during synthesis, ultraviolet irradiation, and surface coatings [27,28]. Michalet X paved the way for precise structural studies of biomolecules and biomolecular complexes by the use of multicolor quantum markers [29]. Gao XH’s research team used subcutaneous injection of QD-labeled cancer cells and systemic injection of multifunctional QD probes to achieve sensitive and multicolor fluorescence imaging of cancer cells under in vivo conditions [30]. Although these scholars were not included in prolific authors, their contributions to QDs toxicity research has had a huge impact on the application of QDs, which will help future researchers to expand their ideas.

As for co-cited references, the co-citation clustering diagram in timeline view indicate that the most influential references were concentrated between 2004 and 2010, suggesting that the research related to QDs is expected to have a significant impact on almost all industries and social fields. Therefore, it is urgent to identify the potentially harmful side-effects of QDs on human health, considering their broad application prospects in various fields [31]. Furthermore, some highly influential papers have also been published in other journals (for example, Nature Biotechnology, Nature Materials, and Langmuir), which will extensively boost QDs toxicity research in the future.

Researchers have found that trends in one field can greatly benefit other scientists and subject development [32]. Burst keywords (abrupt changes or emerging trends) identified with bibliometric analysis promise a rational prediction of research frontiers by reflecting the concerns of some researchers on a series of related research questions and concepts, to some extent [33,34]. Therefore, here, the keywords with the strongest citation bursts were detected and analyzed by the use of CiteSpace V software to understand the development of research on QDs toxicity. Time interval was plotted as the blue line and the period of burst keywords was plotted as the red line, indicating the beginning and end of each burst’s time interval [35]. In this study, we derived the top four frontiers on QDs toxicity research: “stability” (2011–2012), “cadmium” (2011–2015), “*escherichia coli*” (2011–2013), and “walled carbon nanotube” (2012–2015), and the toxicity produced by QDs relevant to the four keywords explained.

Stability: QDs are usually composed of chemical elements in group III-V or II-VI. The core of various QDs contain heavy metal elements, which can cause significant toxicity once released outside [36]. Therefore, the stability of QDs materials is critical because more and more findings from studies suggest the critical influence of stability in the toxicity of QDs. In order to increase stability and promote the biosafety of the QDs, some researchers packed a shell structure (PEG, MPA, et al.) outside the QDs as per different needs to increase the water solubility or bio-melting of the QDs [37]. It was found that QDs with ZnS shell and PEG coating as a protective factor are more beneficial to cell proliferation, when compared to naked QDs [38]. Su et al. [39] researched and compared the cytotoxicity of three kinds of Cd-based QDs (CdTe, CdTe/CdS, and CdTe/CdS/ZnS QDs). The results revealed that CdTe/CdS/ZnS QDs with core-shell-shell structure had the least cytotoxicity, suggesting that the modification of the shell could effectively improve the stability and biocompatibility of QDs as well as inhibit the release of metal ions inside the core. Another research found that ROS release from double-layered thick-shell QDs was about twice that of the corresponding single-shell QDs, confirming the effect of the thick shell on the stability and biosafety of the QDs [40].

Cadmium: Many types of QDs have been developed and prepared by researchers. Among these QDs, cadmium-containing QDs are the most widely applied because of their simple synthesis, high luminous efficiency, excellent monochromaticity, as well as complete coverage of entire visible region by the spectrum [41,42,43]. Furthermore, the current cadmium-containing QDs have certain advantages regarding detection sensitivity in biological applications, which is worthy of attention from researchers. However, cadmium has been confirmed for decades as a toxic heavy metal, so it is no doubt that the toxicity of cadmium-containing QDs is a pioneer topic in the field of nanotoxicology. Since QDs have been reported to be corroded, oxidized, and even dissolved by the microenvironment after entering organisms, many researchers tend to believe that the toxicity of QDs may be related to the leakage of metal ions after the QDs are dissolved. For example, a proton is dissolved and the core shell is separated in a low pH environment such as gastric juice, reducing the stability of QDs and leading to the release of metal ions. As for cadmium-containing QDs, Cd^2+^ is easily released from the core due to biodegradation or photolysis, resulting in the toxicity of QDs [44]. Therefore, it is urgent to find an effective way to ensure the biosafety of cadmium-containing QDs, considering their broad application in many fields [45].

*Escherichiacoli*: With the development of nanotechnology, varieties of QDs with diverse functions had been synthesized by researchers, showing promising applications in many fields due to their superior performance. On the basis of the potential toxicity of these QDs to animals or humans, more and more researchers have used *Escherichiacoli* to evaluate their environmental impact, because this bacteria is one of the most representative species in the rapidly developed nanotoxicity test [46,47,48].

Walled carbon nanotube: Over recent years, researchers have been working to develop QDs using low-toxic or even non-toxic materials since most traditional QDs contain heavy metal ions. Among these novel QDs, carbon QDs can be used as good tracer imaging materials [49,50]. However, the biosafety of these so-called low-toxic QDs has not been fully confirmed by researchers, so the toxicity data from walled carbon nanotube is used to predict and guide the biosafety assessment of carbon QDs [51]. Therefore, toxicological studies on low-toxic QDs have attracted more attention from researchers all over the world.

This study retrieved and collected data on QDs toxicity research publications from the Web of Science Core Collection (WoSCC) database (Science Citation Index-Expanded journal); it is the first bibliometric analysis of the trend on QDs toxicity research in the past decade. The analysis of data was relatively comprehensive and objective. This bibliometric study provided information on QDs toxicity researches—trends, cooperating countries/institutions and authors, journals and papers with reference value, and research frontiers in the field.

However, this study had some limitations. Most of the publications in the WoSCC database were in English. Other databases (such as Scopus, PubMed, and Google Scholar) were not analyzed because WoSCC was more advanced in providing detailed data such as national and institutional information, author information, annual publications, and journal sources. As a result, a large number of high-quality, non-English studies on the toxicity of QDs was not included, making the analysis incomplete. Therefore, future work should include other non-English research works as well.

## 5. Conclusions

Over the last 10 years, researchers have tried their best to develop low-toxic or even non-toxic QDs. The number of published studies on the toxicity of different QDs is increasing in a bid to improve the quantum yield, fluorescence intensity, biocompatibility, and biosafety of QDs. In addition to identifying a continual increase in the number of publications on QDs toxicity research over the past decade, the top journals that contributed most to publications on the topic and the top countries that engaged in the research were found. Meanwhile, good candidates for cooperation and research focus in the next few years were analyzed as well. This study not only provides comprehensive information on the trend of toxicological study of QDs over the past decade, but also predicts pioneered keywords that will guide further research works in the field.

## Figures and Tables

**Table 1:** The top 10 journals that published articles on QDs toxicity research.

Rank	Journal Title	Country	Count	Percent	5-Year IF
1	RSC Advances	England	237	4.37%	3.096
2	Nanoscale	England	178	3.29%	7.713
3	ACS Applied Materials Interfaces	United States	129	2.38%	8.284
4	Biomaterials	England	126	2.33%	9.315
5	Journal of Materials Chemistry B	England	107	1.98%	4.959
6	ACS Nano	United States	96	1.77%	14.82
7	Scientific Reports	England	84	0.016	4.609
8	Nanotechnology	England	83	1.53%	3.467
9	Journal of Nanoscience and Nanotechnology	United States	79	1.46%	1.103
10	Sensors and Actuators B Chemical	Switzerland	73	1.35%	5.118

**Table 2:** The top 10 countries and institutions that contributed to publications on QDs toxicity research.

Rank	Country	Count	Institution	Count
1	Peoples Republic of China	2233	Chinese Acad Sci	368
2	USA	1067	Jilin Univ	125
3	India	603	Soochow Univ	103
4	South Korea	349	Univ Chinese Acad Sci	90
5	Germany	188	Wuhan Univ	89
6	Italy	183	Fudan Univ	83
7	England	177	Shanghai Jiao Tong Univ	83
8	Canada	172	Nanyang Technol Univ	75
9	Japan	162	Southeast Univ	68
10	France	161	Univ Sci Technol China	63

**Table 3:** The top 10 authors, co-cited authors, and co-cited references in QDs toxicity research.

Rank	Author	Count	Co-Cited Author	Count	Co-Cited Reference	Count
1	Liu Y	106	Derfus AM	593	Hardman R, 2006, Environ Health Persp, V114, P165	304
2	Wang J	66	Michalet X	492	Michalet X, 2005, Science, V307, P538	292
3	Zhang Y	61	Gao XH	432	Derfus AM, 2004, Nano Lett, V4, P11	266
4	Wang Y	56	Chan WCW	410	Baker SN, 2010, Angew Chem Int Edit, V49, P6726	222
5	Li Y	48	Medintz IL	388	Choi HS, 2007, Nat Biotechnol, V25, P1165	206
6	Yong KT	46	Hardman R	379	Kirchner C, 2005, Nano Lett, V5, P331	199
7	Zhang J	43	Zhang Y	294	Gao XH, 2004, Nat Biotechnol, V22, P969	190
8	Wang X	38	Choi HS	286	Medintz IL, 2005, Nat Mater, V4, P435	177
9	Yang B	38	Wang Y	277	NEL A, 2006, Science, V311, P622	173
10	Zhang H	36	Bruchez M	274	Cho SJ, 2007, Langmuir, V23, P1974	170

## Data Availability

Publicly available datasets were analyzed in this study. This data can be found in The Web of Science Core Collection (WoSCC).

## Supplementary Materials

The following are available online at https://www.mdpi.com/article/10.3390/ijerph18115768/s1: The terms used for data retrieval.
