# Supplementary material for: Assessment of the Toxicity of Quantum Dots through Biliometric Analysis
Source: Int J Environ Res Public Health. 2021 May 27;18(11):5768. doi: 10.3390/ijerph18115768 (PMC8199113; doi:10.3390/ijerph18115768)
Supplement: Supplementary file 1 [file ijerph-18-05768-s001.zip › ijerph-1181543-supplementary.pdf]

Supplementary Materials

Assessment of the toxicity of quantum dots through a biliometric analysis

Lishi Zhong †1, Lili Zhang †1, Yimeng Li †1, Xue Liang [2], Lu Kong [1], Xiaobing Shen [1], Tianshu Wu [1*]

[1] Key Laboratory of Environmental Medicine and Engineering, Ministry of Education, School of Public Health, Southeast University, Nanjing 210009, Jiangsu, China.

[2] School of Public Health, Shandong First Medical University & Shandong Academy of Medical Sciences, Jinan, P.R.China.

* Correspondence: ninatswu@126..com / ninatswu@seu.edu.cn

† The authors contributed equally to the work.

The terms used for data retrieval:

(1) (TOPIC: (“quantum dot”) or TOPIC: (“quantum dots”) or TOPIC: (“nano dots”) or TOPIC: (“nanocrystals”) or TOPIC: (“nanoscale semiconductor”) or TOPIC: (“QD”) or TOPIC: (“QDs”) or TOPIC: (“QD(s)”) or TOPIC: (“Semiconductor Nanoparticle”) or TOPIC: (“Semiconductor Nanocrystal”)) and (TOPIC: (“toxicity”) or TOPIC: (“toxicology”) or TOPIC: (“toxicological”) or TOPIC: (“toxic”) or TOPIC: (“nanotoxic”) or TOPIC: (“nanotoxicity”) or TOPIC: (“nanotoxicological”) or TOPIC: (“toxicologic”) or TOPIC: (“adverse effect”) or TOPIC: (“adverse health effect”) or TOPIC: (“nanotoxicology”) or TOPIC: (“toxic effect”) or TOPIC: (“poisonous”) or TOPIC: (“poison”) or TOPIC: (“detrimental effect”) or TOPIC: (“survival rate”) or TOPIC: (“health hazard”)).

(2) All references were from 2009 to 2018.

(3) Languages were English.

(4) Literature Types were articles or reviews.
